# Robotic versus laparoscopic low anterior resection for rectal cancer: a meta-analysis

**DOI:** 10.1186/s12957-016-0816-6

**Published:** 2016-03-01

**Authors:** Yanlai Sun, Huirong Xu, Zengjun Li, Jianjun Han, Wentao Song, Junwei Wang, Zhongfa Xu

**Affiliations:** Department of Colorectal Cancer Surgery, Shandong Cancer Hospital and Institute, 440 Jiyan Road, Jinan, 250117 China

**Keywords:** Rectal cancer, da Vinci Surgical System, Laparoscopic surgery, Low anterior resection, Meta-analysis

## Abstract

**Background:**

The objective of this meta-analysis was to compare the clinical and oncologic outcomes of robotic low anterior resection (R-LAR) with conventional laparoscopic low anterior resection (L-LAR).

**Methods:**

A search in the MEDLINE, Embase, and Ovid databases was performed for studies published before July 2014 that compared the clinical and oncologic outcomes of R-LAR and L-LAR. The methodological quality of the selected studies was assessed. Depending on statistical heterogeneity, a fixed or random effects model was used for the meta-analysis. The clinical and oncologic outcomes evaluated included operative time, estimated blood loss, length of hospital stay, rate of conversion to open surgery, post-operative complications, circumferential margin status, and number of lymph nodes collected.

**Results:**

Eight studies, including 324 R-LAR cases and 268 conventional L-LAR cases, were analyzed. The meta-analysis showed that R-LAR was associated with a shorter hospital stay (mean difference (MD) = −1.03; 95 % confidence interval (CI) = −1.78, −0.28; *P* = 0.007), lower conversion rate (odds ratio (OR) = 0.08; 95 % CI = 0.02, 0.31; *P* = 0.0002), lower rate of circumferential margin involvement (OR = 0.5; 95 % CI = 0.25, 1.01; *P* = 0.05), and lower overall complication rate (MD = 0.65; 95 % CI = 0.43, 0.99; *P* = 0.04) compared with L-LAR. There was no difference in operative time (MD = 28.4; 95 % CI = −3.48, 60.27; *P* = 0.08), the number of lymph nodes removed (MD = −0.63; 95 % CI = −0.78, 2.05; *P* = 0.38), and days to return of bowel function (MD = −0.15; 95 % CI = −0.37, 0.06; *P* = 0.17).

**Conclusions:**

R-LAR was shown to be associated with a shorter hospital stay, lower conversion rate, lower rate of circumferential margin involvement, and lower overall complication rate compared with L-LAR. There were no differences in operative time, the number of lymph nodes removed, and days to return of bowel function.

## Background

Laparoscopic colorectal resection has been popularized due to its associated short length of hospital stay, reduced post-operative pain, and early return to normal bowel function [1, 2]; however, the laparoscopic colorectal technique has several drawbacks, such as a two-dimensional view and the limited dexterity of instruments due to the fixed instrument tips [3]. The da Vinci Surgical System was first used by colorectal surgeons in 2002 and was shown to overcome the drawbacks of conventional laparoscopic surgery. The da Vinci Surgical System allows for improved dexterity of movement, 3D and magnified vision, and tremor filtering [4]. Both laparoscopic and robotic surgeries for rectal cancer have been proven to be safe and effective. Thus far, there have been several studies comparing the clinical and oncologic outcomes of robotic versus laparoscopic surgery for rectal cancer [5–7], but few studies and no meta-analyses have been conducted comparing the outcomes of robotic (R-LAR) versus laparoscopic low anterior resection (L-LAR).

## Methods

### Information sources and search

A search in the MEDLINE, Embase, and Ovid databases was performed for studies published before July 2014 comparing clinical or oncologic outcomes of R-LAR and L-LAR. In addition, the abstracts published at major international conferences were manually searched. The following search terms were used: “robotic/robotic-assisted,” “low anterior resection,” “robotic⁄robotic-assisted versus laparoscopic rectal resection,” and “robotic/robotic-assisted versus laparoscopic low anterior resection.”

### Study selection and quality assessment

Two authors (SYL and XHR) obtained full-text articles of relevant studies and independently determined the criteria for inclusion. Disagreements between the two authors were resolved by discussion and consensus. If the negotiation failed, a third independent author (XZF) provided an opinion. The quality of RCTs was evaluated using the Cochrane Reviewer’s Handbook Jadad scale [8], and the quality of the NRCTs was evaluated by the “Methodological Items for Non-randomized Studies” [9].

### Criteria for inclusion and exclusion

The following inclusive criteria were required: (1) randomized or non-randomized studies comparing the clinical and oncologic outcomes of R-LAR and L-LAR; (2) if the same institution and/or authors reported more than one study, the higher quality study was included; (3) studies reported at least one of seven outcomes (operative time, estimated blood loss, length of hospital stay, conversion rate to open surgery, post-operative complications, circumferential margin status, and number of lymph nodes collected); and (4) the definition of the rectal cancer level should be below the peritoneal reflection.

The exclusion criteria were as follows: (1) the clinical and oncologic outcomes were not reported clearly; (2) studies reporting proctectomy for rectal cancer that was not a low anterior resection, such as an abdominoperineal resection and Hartman procedure; (3) overlaps between authors or institutions in the published literature; and (4) studies that lacked control arms.

### Statistical analysis

Review Manager software (RevMan, version 5.2) provided by the Cochrane Collaboration was used to perform the meta-analysis. Continuous variables were pooled using the mean difference (MD) with a 95 % confidence interval (CI), and dichotomous variables were pooled using the odds ratio (OR) with a 95 % CI. If continuous variables were reported as the median with range, we calculated the means and standard deviations according to Hozo [10]. Statistical heterogeneity was evaluated by *I*^2^, and heterogeneity was considered high if the *I*^2^ statistic was >50 %. The fixed effects model was used for studies with low or moderate statistical heterogeneity, and the random effects model was used for studies with high statistical heterogeneity. Sensitivity analysis was performed by repeating the meta-analysis on the studies that were excluded.

## Results

### Eligible studies

**Fig. 1:**
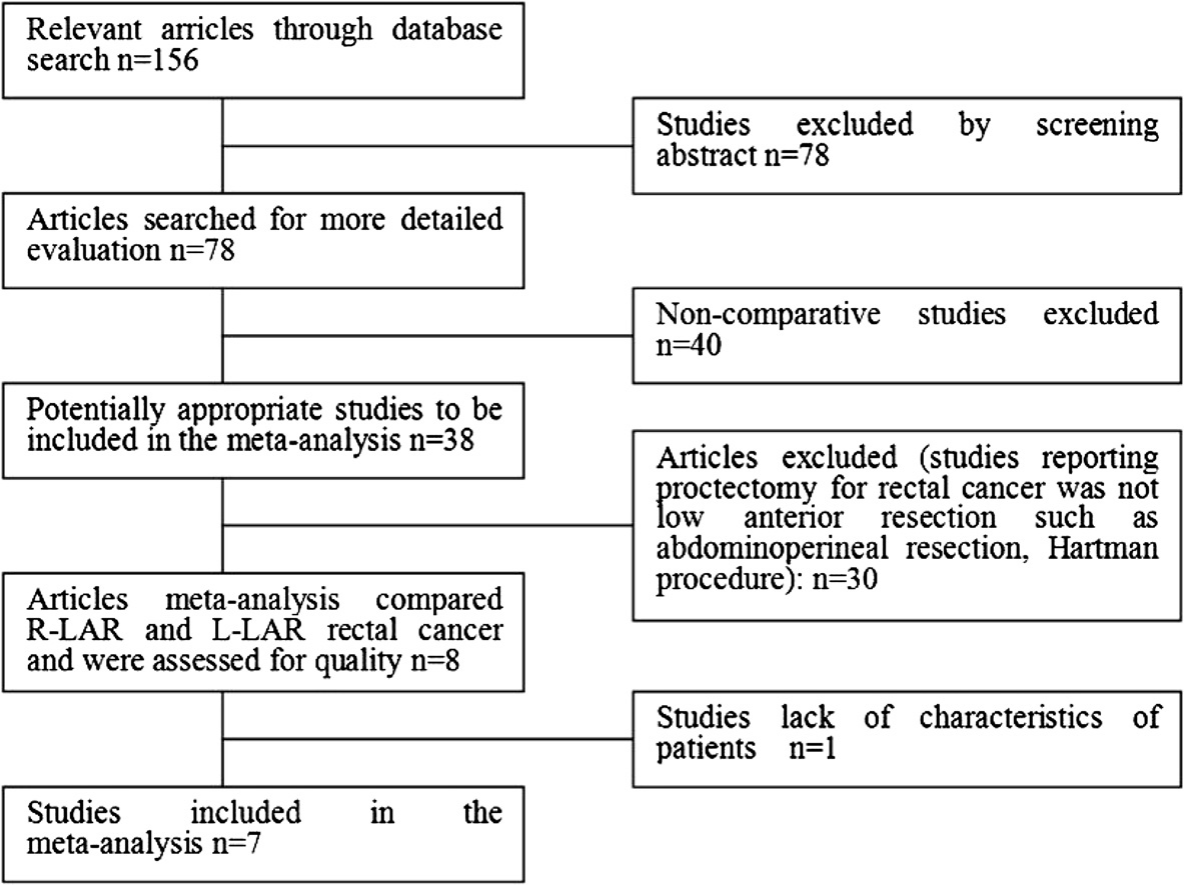
Flow diagram of study selection for meta-analysis

Using the search terms, we initially retrieved 168 publications. After carefully browsing the abstracts and full texts, eight comparative studies [11–18] met all of the inclusion criteria and were eligible for meta-analysis. One comparative study [18] was excluded because it did not include patient characteristics, thus leaving seven suitable studies for the meta-analysis (Fig. 1). The seven studies [11–17] involved a total of 592 patients (324 in the R-LAR group and 268 in the L-LAR group) (Table 1). The seven studies included six non-randomized controlled trials (NRCTs) and one randomized controlled trial (RCT). The characteristics of these seven studies are listed in Table 1. Of all the studies, two were conducted in the USA [12, 17], three in Korea [11, 13, 14], one in Italy [15], and one in Turkey [16]. The quality of all the studies was satisfactory. The results showed that R-LAR had longer operative times, lower estimated blood loss, shorter hospital stays, lower overall post-operative complications, and a significantly faster recovery of bowel function.

**Table 1:** Characteristics of the eight selected studies included in the meta-analysis

Study	Country	Group	Patients	Mean	Mean	Sex	CRT	Study	Anastomosis
				Age	BMI	M/F	(%)	Type	Technique
Park (2015) [11]	Korea	RCC	133	59.2	23.1	86:47	11.3	R	Hybrid
		LRC	84	63.5	22.9	60:24	11.9		
Pigazzi (2006) [12]	USA	RCC	6	60.0	31.0	2:4	33.0	PNR	Total/hybrid
		LRC	6	70.0	27.0	4:2	33.0		
Baik (2008) [13]	Korea	RCC	18	57.3	22.8	14:4	NS	RCT	Hybrid
		LRC	18	62.0	24.0	14:4	NS		
Baik (2009) [14]	Korea	RCC	56	60.3	23.4	37:19	8.9	PNR	Hybrid
		LRC	57	63.2	23.2	34:23	12.3		
Annibale (2013) [15]	Italy	RCC	50	66.0	NS	30:20	68.0	PNR	Total
		LRC	50	65.7	NS	30:20	56.0		
Erguner (2013) [16]	Turkey	RCC	27	54.0	28.3	14:13	14.8	R	Total
		LRC	37	61.5	26.7	20:17	8.0		
Marecik (2011) [17]	USA	RCC	34	60.0	28.5	20:14	58.8	PNR	Hybrid
		LRC	24	64.0	25.9	14:10	41.7		
Shin (2012) [18]	Korea	RCC	30	58.1	22.0	18:12	NS	PNR	Total/hybrid
		LRC	30	63.3	20.0	18:12	NS		

### Operative time

**Fig. 2:**
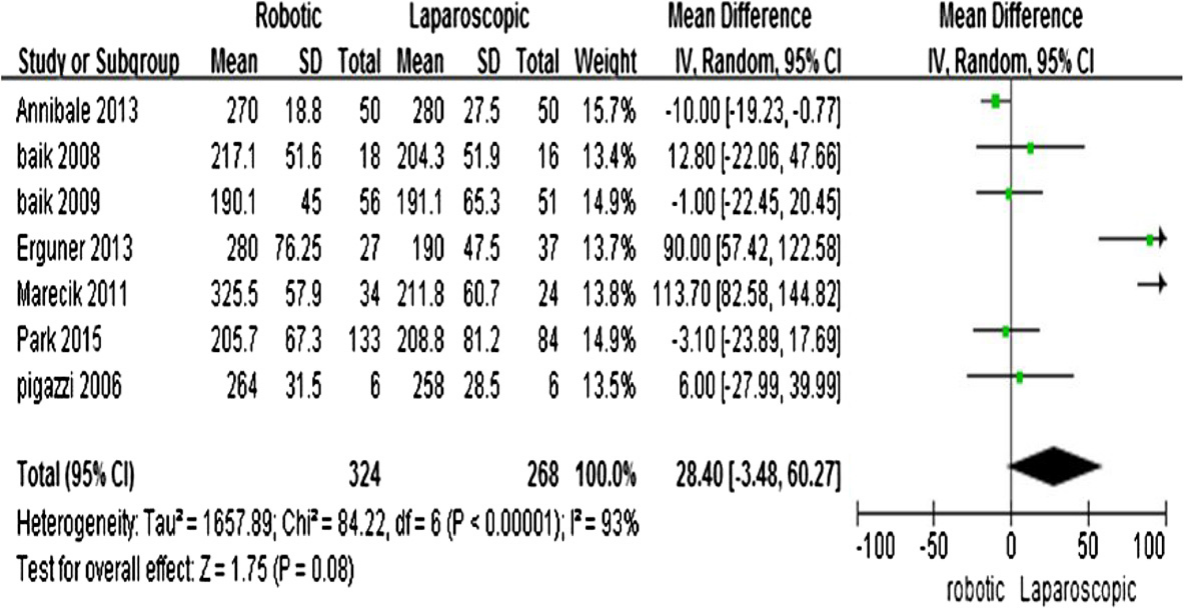
Robotic compared with laparoscopic low anterior resection for cancer: operative time

All of the studies [11–17] reported operative times; the meta-analysis showed no significant difference between the two techniques (MD = 28.4; 95 % CI = −3.48, 60.27; *P* = 0.08). The random effects model was used because of the high heterogeneity among studies (*I*^2^ = 93 %) (Fig. 2).

### Length of hospital stay

**Fig. 3:**
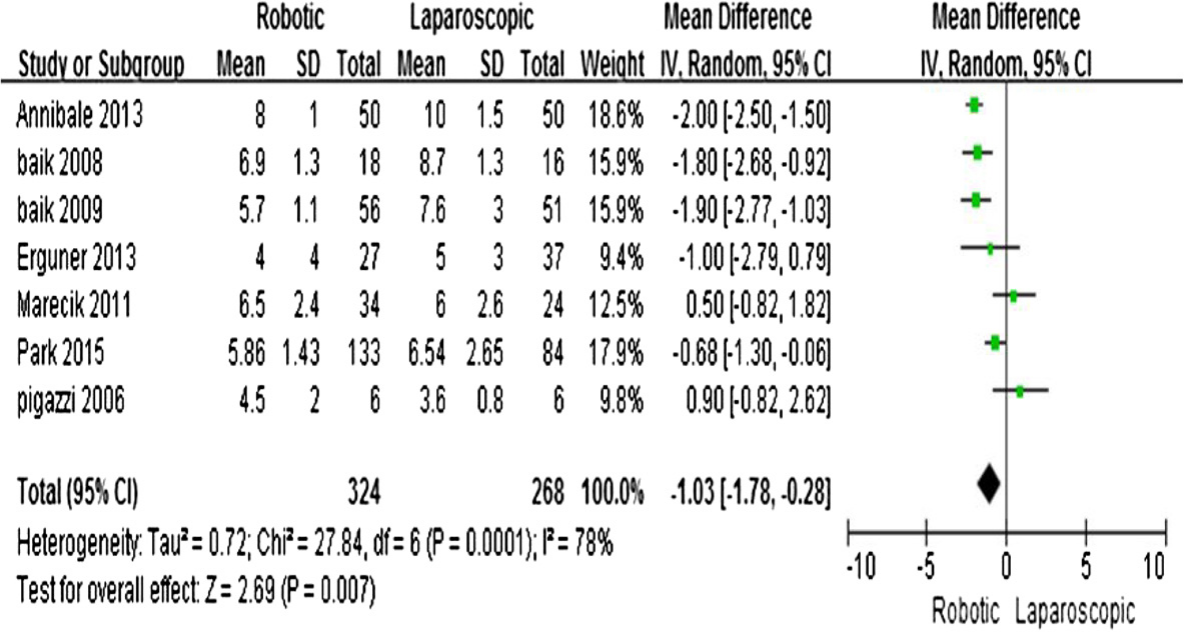
Robotic compared with laparoscopic low anterior resection for cancer: length of hospital stay

All of the studies [11–17] reported the length of hospital stay. The meta-analysis showed that R-LAR required a shorter hospital stay compared with L-LAR (MD = −1.03; 95 % CI = −1.78, −0.28; *P* = 0.007). The heterogeneity was high; therefore, a random effects model was utilized (*I*^2^ = 78 %) (Fig. 3).

### Conversion to open surgery

**Fig. 4:**
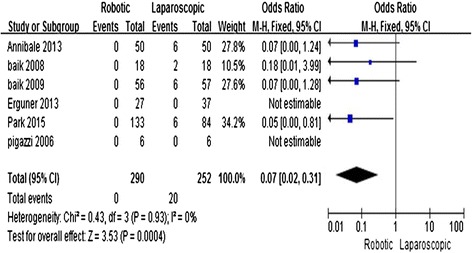
Robotic compared with laparoscopic low anterior resection for cancer: length of hospital stay

Six studies [11–16] reported the rate of conversion to open surgery. The meta-analysis showed that R-LAR had a lower conversion rate compared with L-LAR (OR = 0.07; 95 % CI = 0.02, 0.31; *P* = 0.0004) with no observed heterogeneity (*I*^2^ = 0 %) (Fig. 4).

### Number of harvested lymph nodes

**Fig. 5:**
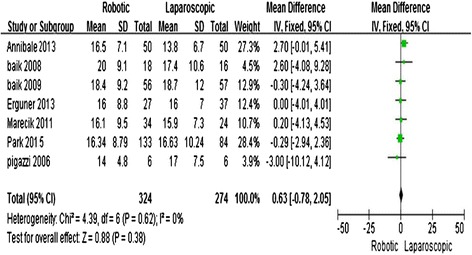
Robotic compared with laparoscopic low anterior resection for cancer: number of harvested lymph nodes

Six studies [11–16] reported the number of harvested lymph nodes. The meta-analysis showed no significant difference between the two techniques (MD = −0.63; 95 % CI = −0.78, 2.05; *P* = 0.38) with no observed heterogeneity (*I*^2^ = 0 %) (Fig. 5).

### Positive circumferential resection margin involvement

**Fig. 6:**
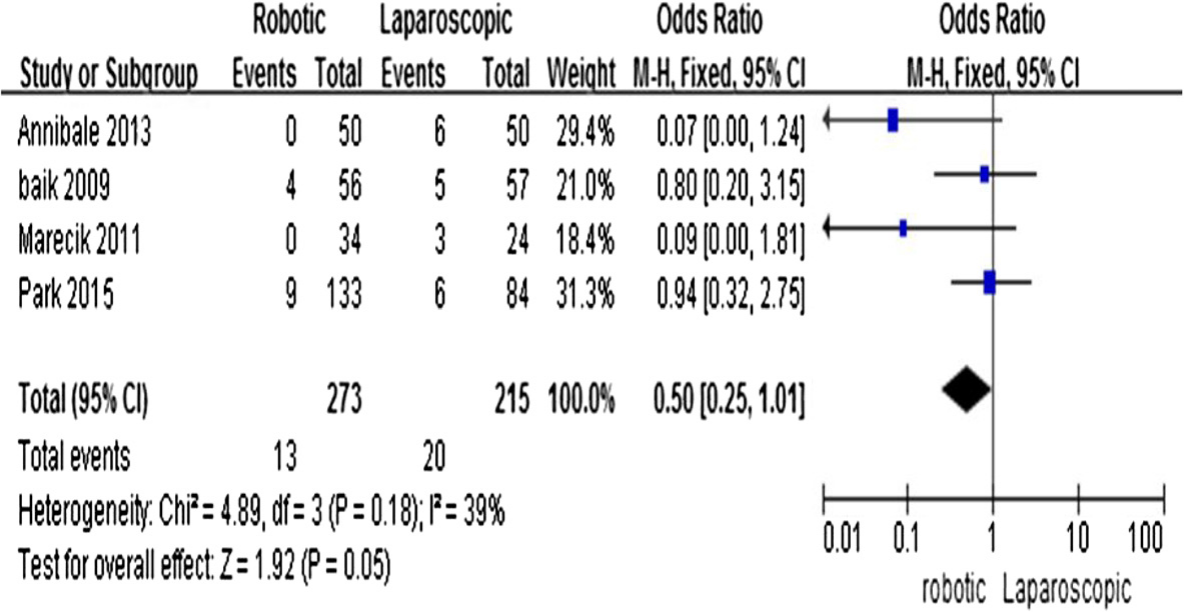
Robotic compared with laparoscopic low anterior resection for cancer: positive circumferential resection margin involvement

Four studies [11, 14, 15, 17] reported the status of the circumferential resection margin (CRM). Circumferential resection margin involvement was found to be significantly lower in the R-LAR group than in the L-LAR group (OR = 0.5; 95 % CI = 0.25, 1.01; *P* = 0.05) with low heterogeneity (*I*^2^ = 39 %) (Fig. 6).

### Post-operative complications

**Fig. 7:**
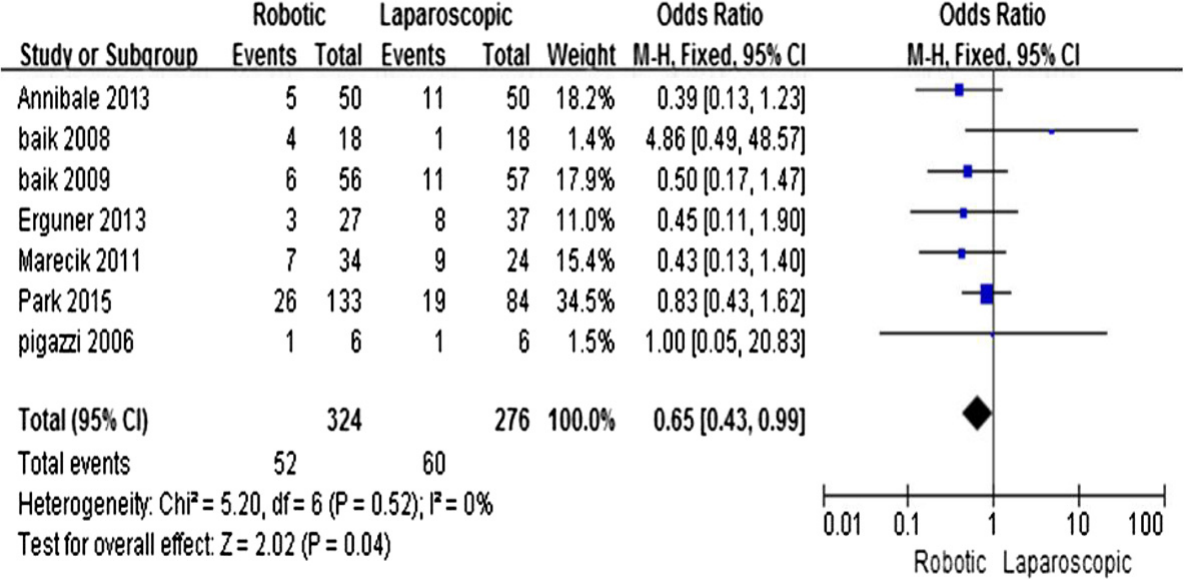
Robotic compared with laparoscopic low anterior resection for cancer: post-operative overall complications

All of the studies [11–17] reported the overall post-operative complication rate. The result of the meta-analysis showed that the overall complication rate was significantly lower in the RRC group (MD = 0.65; 95 % CI = 0.43, 0.99; *P* = 0.04) with no heterogeneity (*I*^2^ = 0 %) (Fig. 7).

### Days to return of bowel function

**Fig. 8:**
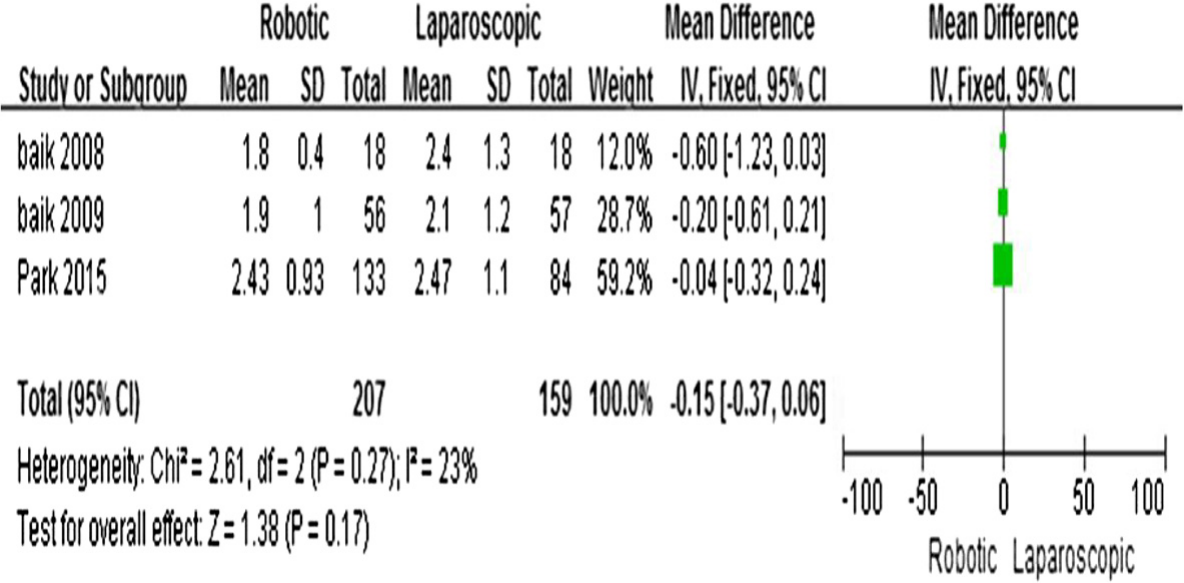
Robotic compared with laparoscopic low anterior resection for cancer: days to return of bowel function

Three studies [11, 13, 14] reported the number of days to passing flatus. The meta-analysis showed no significant difference between the two techniques (MD = −0.15; 95 % CI = −0.37, 0.06; *P* = 0.17) with no observed heterogeneity (*I*^2^ = 23 %) (Fig. 8).

## Discussion

Laparoscopic colorectal surgery was first reported by Jacobs in 1991 [19] and has increasingly become a popular approach for colorectal procedures. The long-term outcomes of laparoscopic colorectal surgery have been shown to be similar to open techniques; however, laparoscopy offers better short-term outcomes [20, 21]. Some of the advantages of the laparoscopic colorectal technique over the open procedures include smaller incisions, reduction in post-operative pain and duration of ileus, quicker post-operative recovery, and earlier return to normal activity [22]. The new meta-analysis indicates that laparoscopy benefits rectal cancer patients with a shorter hospital stay, earlier return of bowel function, reduced blood loss and number of blood transfusions and lower rates of abdominal post-operative bleeding, late intestinal adhesion obstruction, and other late morbidities [23]. The laparoscopic technique still has several limitations, such as tremor, loss of three-dimensional view, poor ergonomics, and limited dexterity of movement due to the fixed instrument tips [24]. The da Vinci Surgical System has overcome these limitations and provides a three-dimensional, high-definition operative field, the steady “traction and counter-traction,” reduces the physiologic tremor, and enables three extra degrees of movement using articulated instruments. However, the da Vinci Surgical System also has drawbacks, such as the loss of haptic feedback, limited range of movement of the robotic arms, time-consuming, and high cost of the system. Several studies [11, 25–27] have compared the clinical and oncologic safety and efficacy of robotic resection and laparoscopic rectal resection for cancer, but no meta-analyses have compared R-LAR with conventional L-LAR. Indeed, this is the first meta-analysis comparing the two approaches.

Although it is better to use RCTs to perform a meta-analysis, randomization is difficult to carry out in surgery. Therefore, NRCTs represent an acceptable alternative when performing a meta-analysis comparing two surgical techniques. We selected studies that we deemed to be of the highest methodological quality; however, selection bias may have existed because most of the studies were non-randomized and pre-operative baseline characteristics were not equal in the included studies. High heterogeneity for some outcomes may have influenced the effect of the meta-analysis; however, the significant impact of factors other than the surgical method affects these outcomes.

The heterogeneity of the length of hospital stay variable was high between studies (*I*^2^ = 78 %). The reason for the high heterogeneity between the groups may be differences in discharge criteria from hospital or different post-operative complications. The three-dimensional, high-definition field of view and augmented dexterity offered by the robotic approach minimizes the risk of injury of tissue and small blood vessels and leads to fewer complications, which may be related to the shorter length of hospital stay. Recently, a systematic review and meta-analysis about possible benefits of robotic surgery regarding urinary and sexual dysfunctions was reported [28], although there were few data and no randomized controlled trials support the results. Looking forward to the future randomized controlled trials could compare this area.

Operative time is considered to be long in robotic colorectal surgery because of additional set-up time, additional docking time, and the steep learning curve associated with this technique [29]. This meta-analysis showed no significant difference in operative time between the two surgical approaches. The heterogeneity in operative time between the two methods was very high (*I*^2^ = 93 %). The set-up time was excluded in all of the studies. The reasons for high heterogeneity of operative time are as follows. (1) Some studies adopt hybrid robotic approaches, while some studies adopt full robotic approaches. (2) Specimen-retrieval techniques (natural orifice or mini-laparotomy) are different. (3) The learning curve in R-LAR is less steep than that in L-LAR procedures, and surgeons are relatively unskilled in the R-LAR technique [30]. As surgeons become more and more adept with the technique, the operative time for R-LAR will decrease.

Local recurrence of rectal cancer after surgery is common and influences survival, and most studies involving CRM focus on local recurrence [31]. The CRM is a powerful prognostic factor for rectal cancer resection. In our study, R-LAR was associated with a lower conversion rate, lower rate of circumferential margin involvement, and lower overall complication rate. These findings may be explained by the advantages of the robotic surgical system, including the three-dimensional operative field, reduction of the physiologic tremor, and the three extra degrees of freedom in movement. In combination, these features may minimize the risk of tissue and vascular injuries. Local recurrence rate, incision recurrence rate, and overall recurrence rate are the key to the success or failure of surgery. In our meta-analysis, few studies focus on the comparison of post-operative recurrence, and it was difficult to perform subgroup analyses. We hope that more randomized controlled studies will be able to compare the post-operative recurrence rate in the future.

The major drawback of robotic surgery for rectal cancer is the cost. Initial purchasing costs, maintenance costs, and equipment costs contribute to the high price. We cannot carry out a comparative analysis of cost because few studies provide related data. A cost-effectiveness analysis should be performed before this approach is widely implemented. Hottenrott [32] reported that the cost may be reduced by increasing productivity and competition. Therefore, this drawback may be overcome as the robotic approach becomes more widely used.

Our meta-analysis had some limitations. First, the meta-analysis only included one RCT and six NRCTs. The latter can bias the interpretation of the results, although the quality of the studies was deemed satisfactory [33]. Second, the number of studies and patients was relatively small, making it difficult to perform a subgroup analysis. Finally, matching patient characteristics is difficult in all of the studies, thus there is still some heterogeneity in the two groups.

## Conclusions

This meta-analysis suggests that R-LAR is associated with a shorter hospital stay, lower conversion rate, lower rate of circumferential margin involvement, and lower overall complication rate than L-LAR. There were no differences in operative time, the number of lymph nodes removed, and the number of days to return of bowel function. At present, there are larger randomized controlled trials (trial ROLARR) comparing robotic versus laparoscopic rectal resection, and we hope to have more randomized controlled studies comparing robotic and laparoscopic low anterior resection.

## Abbreviations

BMI: body mass index
CRM: circumferential resection margin
L-LAR: laparoscopic low anterior resection
MD: mean difference
MeSH: medical subject headings
NRCTs: non-randomized controlled trials
OR: odds ratio
RCT: randomized controlled trial
R-LAR: robotic low anterior resection
